# Hybrid leader based optimization: a new stochastic optimization algorithm for solving optimization applications

**DOI:** 10.1038/s41598-022-09514-0

**Published:** 2022-04-01

**Authors:** Mohammad Dehghani, Pavel Trojovský

**Affiliations:** Department of Mathematics, Faculty of Science, University of Hradec Králové, Rokitankého, 62, 50003 Hradec Králové, Czech Republic

**Keywords:** Engineering, Mathematics and computing

## Abstract

In this paper, a new optimization algorithm called hybrid leader-based optimization (HLBO) is introduced that is applicable in optimization challenges. The main idea of HLBO is to guide the algorithm population under the guidance of a hybrid leader. The stages of HLBO are modeled mathematically in two phases of exploration and exploitation. The efficiency of HLBO in optimization is tested by finding solutions to twenty-three standard benchmark functions of different types of unimodal and multimodal. The optimization results of unimodal functions indicate the high exploitation ability of HLBO in local search for better convergence to global optimal, while the optimization results of multimodal functions show the high exploration ability of HLBO in global search to accurately scan different areas of search space. In addition, the performance of HLBO on solving IEEE CEC 2017 benchmark functions including thirty objective functions is evaluated. The optimization results show the efficiency of HLBO in handling complex objective functions. The quality of the results obtained from HLBO is compared with the results of ten well-known algorithms. The simulation results show the superiority of HLBO in convergence to the global solution as well as the passage of optimally localized areas of the search space compared to ten competing algorithms. In addition, the implementation of HLBO on four engineering design issues demonstrates the applicability of HLBO in real-world problem solving.

## Introduction

Advances in science and technology have led to the emergence of new optimization challenges as well as the complexity of optimization problems. These cases indicate the need and importance of optimization with efficient tools to achieve optimal solutions. An optimization problem is identified and modeled with three main parts: decision variables, constraints, and objective function^1^. The goal in optimization is to achieve the best solution with respect to the constraints of the problem, among all solutions defined for an optimization problem^2^. Problem solving techniques in optimization applications fall into two groups of deterministic methods and stochastic methods. Deterministic methods using derivative information have acceptable performance in linear and convex spaces. However, these methods are incapable of dealing with high dimension and constraint problems, complex objective functions, nonlinear and non-convex spaces. Stochastic methods, by employing random operators and random scanning of the search space away from the difficulties of deterministic methods, have the ability to provide acceptable solutions to optimization problems. Simplicity in understanding, ease of implementation, no need for derivative information, the ability to cross local optimal areas, applicability in nonlinear, and non-convex spaces are some of the advantages that have led to the popularity and pervasiveness of random methods. Optimization algorithms are one of the most popular techniques in the stochastic approach to optimizing the problem^3^. How to achieve the solution in optimization algorithms begins with generating a certain number of candidate solutions (equal to the population of the algorithm). Evaluating the objective function of the problem based on these candidate solutions determines the quality of each solution. Using this information and the algorithm steps, these candidate solutions are improved in an iterative process. Once the algorithm is fully implemented, the best candidate solution that provides a better value for the objective function compared to other candidate solutions is identified. Given the fact that every optimization problem has a basic solution called global optimal, the point made in optimization studies is that optimization algorithms do not guarantee that they can achieve exactly the global optimal solution. Therefore, quasi-optimal is the name given to the solutions obtained from the optimization algorithms^4^. Efforts to reduce the differences between quasi-optimal solutions and global optimal solutions to find better solutions have paved the way for the design and development of numerous optimization algorithms.

Exploration and exploitation are capabilities that enable optimization algorithms to be efficient in finding solutions. Exploration is the ability to search globally in different areas of the search space while exploitation is the ability to search locally near the solutions obtained because there may be better solutions near those solutions. Balancing exploration and exploitation play a key role in the success of optimization algorithms in achieving optimal solutions^5^. The main research question in the study of optimization algorithms is whether there is still a need to introduce new optimization algorithms despite the fact that countless algorithms have been introduced so far. The No Free Lunch (NFL) theorem^6^ answers this question. The concept of the NFL theorem explains that there is no guarantee that an algorithm with optimal performance in solving a set of objective functions and problems will be able to perform the same performance in all optimization applications. It is not possible to ensure that a particular algorithm is the best optimizer in all optimization topics. The NFL theorem encourages researchers to develop new algorithms to find better solutions to optimization problems. The NFL theorem has motivated researchers in this paper to develop a new optimization algorithm for optimization applications.

Innovation of this study is in introducing and designing a new evolutionary algorithm called Hybrid Leader Optimization (HLBO). The main contributions of this paper are as follows:

- A new stochastic-based optimization algorithm is presented, whose fundamental idea is to guide the population algorithm based on a hybrid leader generated by three different members.
- The stages of HLBO are described in two phases of exploration and exploitation and are mathematically modeled.
- The efficiency of HLBO has been benchmarked by optimizing twenty-three objective functions of a variety of unimodal and multimodal types.
- To evaluate the capability of HLBO, its performance has been compared with ten well-known algorithms.

In this section and in the following section, the related works are presented. The Hybrid Leader Optimization (HLBO) algorithm is introduced and modeled in the section “Hybrid Leader-Based Optimization”. Simulation studies are included in the section “Simulation Studies and Results”. The discussion of HLBO results is provided in the section “Results and Discussion”. HLBO performance test on IEEE CEC 2017 is presented in “Evaluation of the Effectiveness of HLBO in Handling Complex IEEE CEC 2017 Objective Functions”. Conclusions and several research subjects are provided for further study in the last section.

## Related works

Optimization algorithms are stochastic techniques to solve optimization applications that are based on the concepts of stochastic mechanisms, e.g., concretely on random methods of trial and error, modeling of natural processes, animal behavior, physical sciences, biology sciences, rules of games and other evolutionary processes^7^. The main idea applied in the design categorizes the optimization algorithms into five groups: evolutionary-based, swarm-based, physics-based, game-based, and human-based optimization algorithms.

Evolutionary-based algorithms have been developed using the concept of natural selection, the concepts of biological and genetic sciences, and random operators such as selection, crossover, and mutation. Genetic Algorithm (GA)^8^ and Differential Evolutionary (DE)^9^ are the most significant evolutionary algorithms whose main inspiration is modeling of the reproductive process. Simulation of the human immune system against diseases has paved the way for the design of an Artificial Immune System (AIS) algorithm^10^.

Swarm-based algorithms are inspired by the behaviors and strategies of animals, insects, birds, and other swarming activities in nature. The most widely used and famous techniques of this group are Particle Swarm Optimization (PSO)^11^, Ant Colony Optimization (ACO)^12^, Artificial Bee Colony (ABC)^13^, Firefly Algorithm (FA)^14^. The strategy of birds and fish in finding food sources using individual and collective information has been the basic inspiration in designing PSO. The ACO’s main idea has been the ability of ant colonies to find the shortest path between the nest and the food source, taking advantage of its pheromone properties and accumulation. Utilizing the collective intelligence and smart behavior of the bee colony to search and find food has been the fundamental inspiration in ABC design. The light emitted by fireflies can be used for a variety of reasons, such as attracting prey and hunting, attracting other members of the group (attracting the opposite sex), and as a communication strategy. This fascinating light of fireflies has been a remarkable and interesting phenomenon, the inspiration of which has led to the development of the FA.

Searching strategies and behaviors of animals, birds, and insects to find food sources or prey hunting have been the main ideas in the design of various techniques such as Grey Wolf Optimization (GWO) algorithm^15^, Pelican Optimization Algorithm (POA)^16^, Marine Predator Algorithm (MPA)^17^, Orca Predation Algorithm (OPA)^18^, Whale Optimization Algorithm (WOA)^19^, which numerous efforts to improve it have led to “enhanced WOA” versions^20,21^, Reptile Search Algorithm (RSA)^22^, and Tunicate Search Algorithm (TSA)^23^.

Other swarm-based algorithms include Hunger Games Search (HGS)^24^, slime mould algorithm (SMA)^25^], Farmland Fertility^26^, African Vultures Optimization Algorithm (AVOA)^27^, Artificial Gorilla Troops Optimizer (GTO)^28^, Butterfly Optimization Algorithm^29^, Symbiotic Organisms Search (SOS)^30^, Tree Seed Algorithm (TSA)^31^, and Spotted Hyena Optimizer (SHO)^32^.

Physics-based algorithms have been developed on the base of using some physical processes and modeling of physical forces and laws. Simulated Annealing (SA) is the name of the most familiar physics-based algorithm based on simulation of the cooling of a molten metal in the refrigeration process^33^. The use of gravity force along with Newton’s laws of motion have been the basic principles employed in Gravitational Search Algorithm (GSA) design^34^. Flow regimes and classical fluid mechanics have been a fundamental inspiration in developing Flow Regime Algorithm (FRA)^35^. Mathematical modeling of the nuclear reaction process in two stages of nuclear fusion and nuclear fission is employed in the design of Nuclear Reaction Optimization (NRO)^36^. The application of three concepts in cosmology, including wormholes, black holes, and white holes, has been the basis of the Multi-Verse Optimizer (MVO) design^37^.

Game-based algorithms are inspired by player behaviors, rules governing individual and group games. The strategy used by different players to put the puzzle pieces together and solve it has been the idea of designing the Puzzle Optimization Algorithm (POA)^38^. Simulation of the coaching process, holding competitions, and teams interacting with each other during a competitive season of volleyball has led to the design of the Volleyball Premier League (VPL) optimization method^39^. Mathematizing the competition between teams and groups playing a tug-of-war game and trying to win has been the main idea in the development of Tug of War Optimization (TWO) approach^40^.

Human-based algorithms are developed based on the simulation of human activities and behaviors in performing various tasks. Among the approaches of this group can be mentioned Teaching-Learning-Based Optimization (TLBO) based on modeling the interactions of a teacher and learners in the classroom^41^, Poor and Rich Optimization (PRO) based on the modeling of the efforts of the rich and poor groups to improve their economic situation^42^, and Human Behavior-Based Optimization (HBBO) based on the modeling of human thoughts and behaviors^43^.

Scientists’ research in optimization studies also includes improving existing algorithms^44–47^, extending hybrid algorithms by combining different algorithms to increase their efficiency^48,49^, and developing binary versions of optimization algorithms^50–53^.

## Hybrid leader-based optimization

In this section, the concepts of the proposed Hybrid Leader-Based Optimization (HLBO) approach are stated and the HLBO mathematical formulation is presented.

### Inspiration and main idea of HLBO

In population-based algorithms, each member of the population is a searcher in the problem-solving space and therefore a candidate solution. Based on the algorithm steps and information transfer, the population members are able to improve their position to provide better solutions. The dependence of the algorithm population update process on specific members (such as the best member of the population and the worst member of the population) may prevent the algorithm from searching globally in the problem-solving space. These conditions can lead to the rapid convergence of the algorithm towards the local optimal solution and as a result, the algorithm fails to identify the main optimal area in the search space. Therefore, overreliance on the process of updating the algorithm population to certain members reduces the exploration ability within the algorithm. In the proposed HLBO method, a unique hybrid leader is employed to update and guide each member of the algorithm population in the search space. This hybrid leader is generated based on three different members including the best member, one random member, and the corresponding member.

### Mathematical model of HLBO

The HLBO population is similar to other population-based algorithms that can be mathematically modeled using a matrix according to Eq. (1).

1 $$\begin{aligned} X = \begin{bmatrix} X_1 \\ \vdots \\ X_i \\ \vdots \\ X_N \end{bmatrix}_{N\times m} = \begin{bmatrix} x_{11} &{} \cdots &{} x_{1j} &{} \cdots &{} x_{1m} \\ \vdots &{} \ddots &{} \vdots &{} \ddots &{} \vdots \\ x_{i1} &{} \cdots &{} x_{ij} &{} \cdots &{} x_{1m} \\ \vdots &{} \ddots &{} \vdots &{} \ddots &{} \vdots \\ x_{N1} &{} \cdots &{} x_{Nj} &{} \cdots &{} x_{Nm} \\ \end{bmatrix}_{N\times m}~, \end{aligned}$$

where *X* is the HLBO population, $X_i$ is the *i*th candidate solution, $x_{i,j}$ is the value of *j*th variable determined by the *i*th candidate solution, *N* is the size of HLBO population, and *m* is the number of problem variables.

The position of each member $X_i$, $i=1,2,\dots ,N$, of the population *X* is initially initialized randomly by considering the constraints of the problem variables based on Eq. (2).

2 $$\begin{aligned} x_{i,j} = lb_j + r\cdot (ub_j-lb_j) , \ j=1,2,\dots m, \end{aligned}$$

where *r* is a random real number from the interval [0, 1], $lb_j$ and $ub_j$ are the lower and upper bound of the *j*th problem variables respectively.

The objective function of the problem is evaluated based on each of the candidate solutions determined by the members of the population *X*, which is specified in Eq. (3) using a vector.

3 $$\begin{aligned} {\mathbb {F}} = \begin{bmatrix} F_1 \\ \vdots \\ F_i \\ \vdots \\ F_N \end{bmatrix}_{N\times 1} = \begin{bmatrix} F(X_1) \\ \vdots \\ F(X_i) \\ \vdots \\ F(X_N) \end{bmatrix}_{N\times 1}~, \end{aligned}$$

where ${\mathbb {F}}$ represents the vector of the objective functions and $F_i$ denotes the objective function value delivered from the *i*th candidate solution.

The values obtained for the objective function are a measure of the quality of the candidate solutions. The member that provides the best value for the objective function is known as the best member $(X_{best})$ and the member that provides the worst value for the objective function is known as the worst member $(X_{worst})$. These values are updated in each algorithm iteration. What distinguishes optimization algorithms from each other is the process used to update the algorithm population. Two important and influential indicators in the performance of optimization algorithms that should be considered in the process of updating and changing the position in the search space are exploration (global search) and exploitation (local search).

#### Phase 1: Exploration (global search)

Exploration is a feature that enables members of the algorithm population to accurately scan different areas of the search space to be able to find the original optimal area. Excessive reliance on specific members of the population (such as the best member) in the process of updating the algorithm population position prevents the global search of the algorithm in the search space and reduces the algorithm’s ability to explore. This dependence in the update process can lead to early convergence of the algorithm to the local optimal and as a result the algorithm fails to identify the main optimal area in the search space. However, some population members, like the best member, have useful information that should not be overlooked. HLBO uses a hybrid leader to update members of the population. This hybrid leader is produced for each member of the population at each repetition. In constructing a random leader, three members of the population, including

(i) the corresponding member (the same member to be led by this hybrid leader),
(ii) the best member,
(iii) a random member of the population is influential.

The participation coefficient of each of these three members in the production of the hybrid leader is based on the quality of that member in providing a better value for the objective function. The quality of each member of the population in presenting the candidate solution is calculated using Eq. (4).

4 $$\begin{aligned} q_i = \frac{F_i-F_{worst}}{\sum _{j=1}^N (F_j-F_{worst})}~, \quad i\in \{1,2,\dots N\}. \end{aligned}$$

Then, using the results of Eq. (4), the participation coefficients for each member are calculated using Eq. (5).

5 $$\begin{aligned} PC_i = \frac{q_i}{q_i+q_{best}+q_k}~, \qquad \qquad PC_{best} = \frac{q_{best}}{q_i+q_{best}+q_k}~, \qquad \qquad PC_k = \frac{q_k}{q_i+q_{best}+q_k}, \end{aligned}$$

where $i,k \in \{1,2,\dots ,N\}$, $k\not =i$, $q_i$ is the quality of the *i*th candidate solution, $F_{worst}$ is the value of the objective function of the worst candidate solution, $PC_i$, $PC_{best}$, $PC_k$ are the participation coefficients of the *i*th member, the best member, and the *k*th member (*k* is an integer determined randomly from the set $\{1,2,\dots ,N\}$), respectively, in producing the hybrid leader.

After determining the participation coefficients, the hybrid leader is generated for each member of the population using Eq. (6).

6 $$\begin{aligned} HL_i = PC_i\cdot X_i + PC_{best} \cdot X_{best} + PC_k \cdot X_k~, \end{aligned}$$

where $HL_i$ is the hybrid leader for the *i*th member and $X_k$ is a randomly selected population member which the index *k* is the row number of this member in the population matrix. The new position for each member of the population in the search space under the guidance of the hybrid leader is calculated using Eq. (7). This new position is acceptable to the corresponding member if the value of the objective function is improved from the previous position, otherwise it remains in the previous position. These update conditions are modeled in Eq. (8).

7 $$\begin{aligned} x_{i,j}^{new,P1}= & {} {\left\{ \begin{array}{ll} x_{i,j} + r\cdot (HL_{i,j} + I\cdot x_{i,j} )~, &{} F_{HL_i} < F_i; \\ x_{i,j} + r\cdot (x_{i,j} - HL_{i,j} ), &{} \text {else},\\ \end{array}\right. } \end{aligned}$$

8 $$\begin{aligned} X_{i}= & {} {\left\{ \begin{array}{ll} X_{i}^{new,P1}, &{} F_{i}^{new,P1} < F_i; \\ X_{i}, &{} \text {else}, \\ \end{array}\right. } \end{aligned}$$

where $X_i^{new,P1}$ is the new position of the *i*th member, $x_{i,j}^{new,P1}$ is its *j*th dimension, $F_i^{new,P1}$ is its objective function value based on the first phase of HLBO, *r* is a random real number from the interval [0, 1], *I* is an integer which is selected randomly from the set $\{1,2\}$, and $F_{HL_i}$ is the value of the objective function obtained from hybrid leader of the *i*th member.

#### Phase 2: Exploitation (local search)

Exploitation is an ability for members of the algorithm population that enables them to search locally for finding better solutions near the obtained solutions. Therefore, in HLBO a neighborhood around each member of the population is considered that allows that member to change position by searching locally in that area and finding a position with a better value for the objective function. This local search is modeled to improve and increase HLBO exploitation ability using Eq. (9). In this phase, the newly calculated position is also acceptable if it improves the value of the objective function, which is simulated in Eq. (10).

9 $$\begin{aligned} x_{i,j}^{new,P2}= & {} x_{i,j} + (1-2r)\cdot R\cdot \left( 1 - \frac{t}{T} \right) \cdot x_{i,j}, \end{aligned}$$

10 $$\begin{aligned} X_{i}= & {} {\left\{ \begin{array}{ll} X_{i}^{new,P2} ~, &{} F_{i}^{new,P2} < F_i; \\ X_{i}~, &{} \text {else}, \\ \end{array}\right. } \end{aligned}$$

where $X_i^{new,P2}$ is the new position of the *i*th member, $x_{i,j}^{new,P2}$ is its *j*th dimension, $F_i^{new,P2}$ is its objective function value based on the second phase of HLBO, *R* is the constant equal to 0.2, *t* is the iteration counter, and *T* is the maximum number of iterations.

#### Repetition process, pseudo-code, and flowchart of HLBO

**Figure 1:**
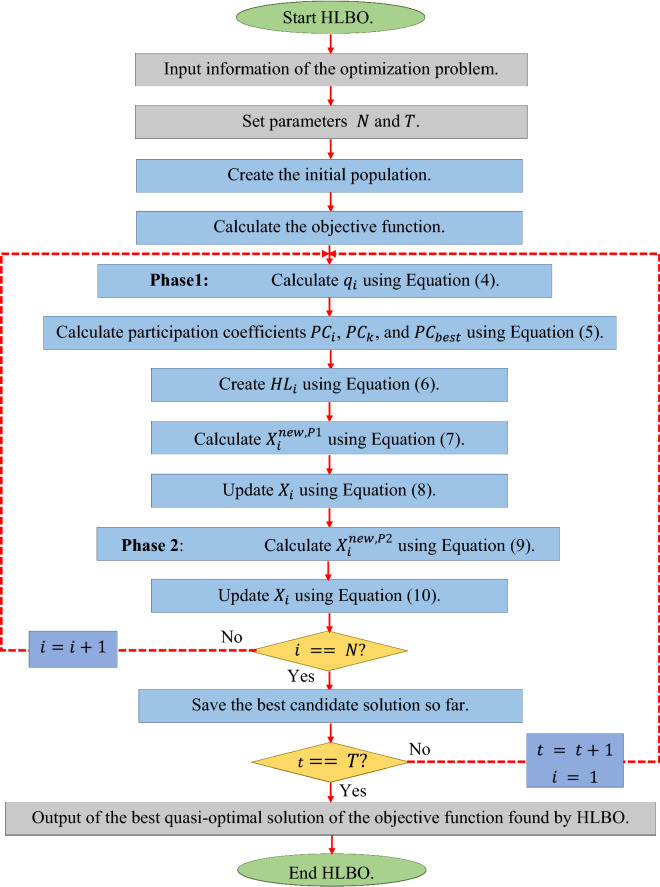
Convergence curves of the HLBO and competitor algorithms in optimizing objective functions $F_1$ to $F_{23}$.

By implementing the first and second phases, all HLBO members are updated and an iteration of the algorithm is completed. The algorithm enters the next iteration and the HLBO population update process continues based on the exploration and exploitation phases according to Eqs. (4)–(10). This process continues until the end of the algorithm, and finally the best candidate solution experienced during the iterations is introduced as the solution to the problem. The HLBO pseudocode is presented in Algorithm 1 and its flowchart is presented in Fig. 1. 
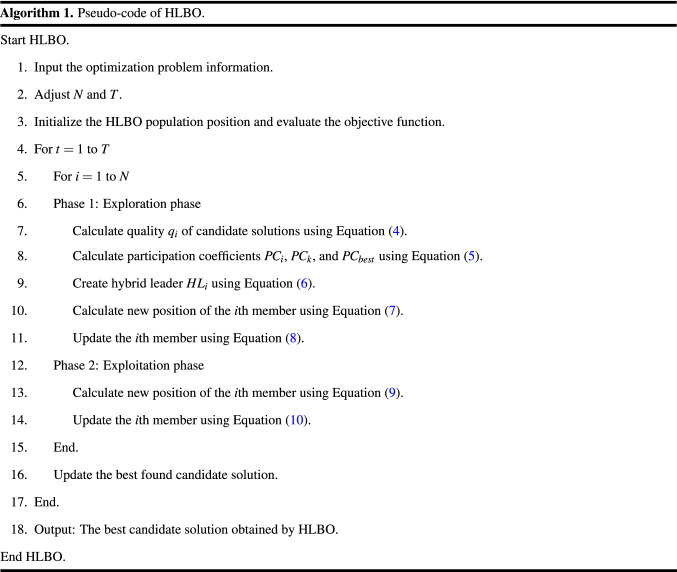

## Computational complexity of HLBO

The HLBO initialization and preparation process has a computational complexity equal to $O(N\,m)$, where *N* refers to the number of population members and *m* is the number of variables in the problem. In each iteration, for each member, a hybrid leader must be generated, resulting in the computational complexity of generating the hybrid leaders equal to $O(N\,m\,T)$, where *T* is the maximum number of iterations of the algorithm. The HLBO update process has two phases of exploration and exploitation, which in both phases the objective function is evaluated. As a result, the computational complexity of HLBO update process equals $O(2N\,m\,T)$. Thus, the total computational complexity of HLBO is equal to $O(N\,m(1+3T))$.

### Simulation studies and results

This section is devoted to simulation studies and evaluation of the proposed HLBO performance in optimization. HLBO has been implemented to provide optimal solutions of twenty-three standard benchmark functions of three main types (complete definitions, domains, and tables of suitable values of parameters of functions $F_1$ to $F_{23}$ can be found in the paper^54^) unimodal function (functions $F_1$ to $F_7$), high-dimensional multimodal functions (functions $F_8$ to $F_{13}$), and fixed-dimensional multimodal functions (functions $F_1$ to $F_7$). The optimization results obtained from HLBO are compared with the performance of ten well-known algorithms including PSO, MPA, HGS, SMA, GA, WOA, TLBO, TSA, GSA, and GWO. The HLBO and the ten mentioned algorithms in twenty independent implementations are employed in optimizing the benchmark functions while each iteration contains 1000 iterations. The optimization results are reported using four statistical indicators: mean, best, standard deviation, and median. Moreover, the rank of each algorithm in providing a better solution for each benchmark function as well as for each group of objective functions is specified. Table 1 lists the adjusted values of the control parameters of the ten competitor algorithms.

**Table 1:** Adjusted values of the control parameters of ten competitor algorithms.

Algorithm	Parameter	Value
HGS	Ranging controller	*R* is gradually reduced to 0
		$$R=2\cdot shrink \cdot rand - shrink$$
		$$shrink=2(1-t/T)$$
SMA	Random parameter	$Z = 0.03$
MPA	Binary vector	$U = 0$ or $U = 1$
	Random vector	*R* is a vector of uniform random numbers in [0, 1]
	Constant number	$P = 0.5$
	Fish Aggregating Devices	$FADs=2$
TSA	$c_1, c_2, c_3$	Random numbers, which lie in the interval [0, 1]
	*Pmin*	1
	*Pmax*	4
WOA	*l* is a random number in $[-1,1]$	
	*r* is a random vector in [0, 1]	
	Convergence parameter *a*	*a*: Linear reduction from 2 to 0
GWO	Convergence parameter *a*	*a*: Linear reduction from 2 to 0
TLBO	random number	*rand* is a random number from the interval [0, 1]
	$T_F$: teaching factor	$T_F = \text {round}\, (1+rand)$
GSA	*Alpha*	20
	$G_0$	100
	*Rnorm*	2
	*Rnorm*	1
PSO	Velocity limit	10% of dimension range
	Topology	Fully connected
	Inertia weight	Linear reduction from 0.9 to 0.1
	Cognitive and social constant	$(C_1,C_2 )=(2,2)$
GA	Type	Real coded
	Mutation	Gaussian ($Probability = 0.05$)
	Crossover	Whole arithmetic ($Probability = 0.8$)
	Selection	Roulette wheel (proportionate)

### Evaluation of unimodal benchmark functions

The results of optimization of $F_1$ to $F_7$ benchmark functions using HLBO and competitor algorithms are released in Table 2. Experimental results show that HLBO provides the global optimal for $F_1$ and $F_6$. HLBO is the best optimizer against competitor algorithms in optimizing $F_2$, $F_4$, and $F_7$. HLBO ranks as the second in $F_3$ optimization and the third in $F_5$ optimization. What can be deduced from the analysis of the reported results is that HLBO is highly efficient in addressing unimodal optimization problems compared to ten competitor algorithms.

### Evaluation of high-dimensional multimodal benchmark functions

The employment results of HLBO and ten competitor algorithms in optimizing $F_8$ to $F_{13}$ benchmark high-dimensional multimodal functions are reported in Table 3. HLBO has managed to find the global optimum by optimizing the functions $F_9$ and $F_{11}$. HLBO is the first best optimizer for handling the function $F_{10}$. In the case of the functions $F_{12}$ and $F_{13}$ the algorithm HGS is the first best optimizer, respectively, while HLBO is the fourth best optimizer for these functions. Analysis of simulation results shows HLBO capability in solving high-dimensional multimodal optimization problems.

### Evaluation of fixed-dimensional multimodal benchmark functions

The results of implementing HLBO and competitor algorithms on benchmark $F_{14}$ to $F_{23}$ benchmark functions are presented in Table 4. What is evident from the simulation results is that HLBO is the first best optimizer in solving $F_{14}$ to $F_{23}$ benchmark functions compared to competitor algorithms. The presented experimental results show that HLBO has a superior performance over similar algorithms in dealing with multimodal optimization problems.

**Figure 2:**
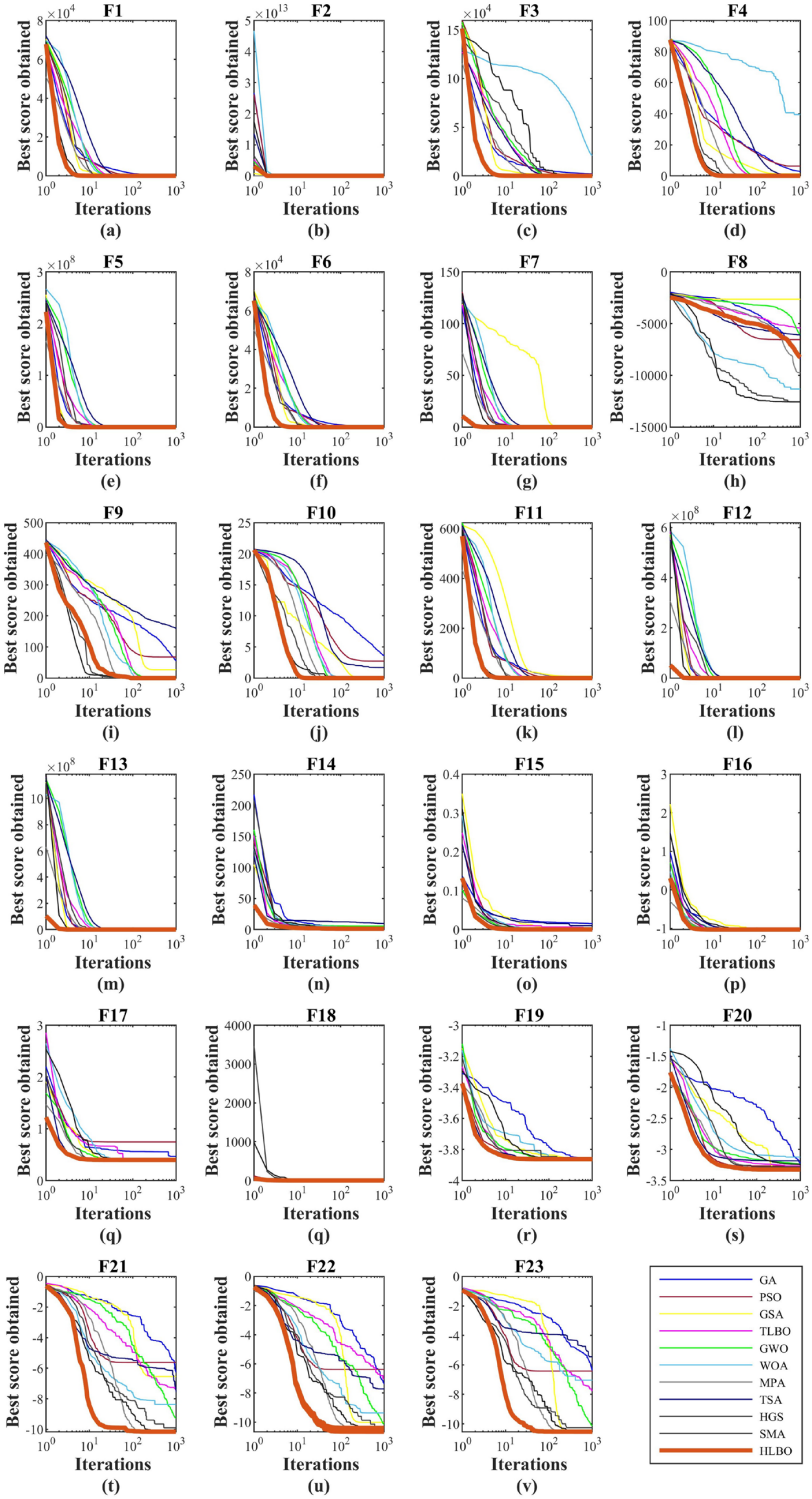
Convergence curves of the HLBO and competitor algorithms in optimizing objective functions $F_1$ to $F_{23}$.

The behavior of convergence curves of HLBO and competitor algorithms in achieving solutions for objective functions $F_1$ to $F_{23}$ is presented in Fig. 2.

### Statistical analysis

In this subsection, by using statistical analysis on the obtained optimization results, the superiority of HLBO over competitor algorithms is examined from a statistical point of view to determine whether this superiority is significant or not. Wilcoxon sum rank test^55^ is employed to address this goal. In this test, an index called p-value indicates and determines the superiority of the target algorithm over the competitor alternative algorithm. The Wilcoxon simulation results are released in Table 5. What can be deduced from the simulation findings is that HLBO has a significant statistical superiority over the competitor algorithm in cases with *p*-values less than 0.05.

## Evaluation of the effectiveness of HLBO in handling complex IEEE CEC 2017 objective functions

In the previous section, the performance of HLBO in handling the objective and multimodal target functions was examined, indicating the satisfactory results of the proposed approach. In this section, the effectiveness of HLBO in solving complex IEEE CEC 2017 benchmark functions^56^ is evaluated. The implementation results of HLBO as well as ten competitor algorithms on objective functions $C_1$ to $C_{30}$ are presented in Tables 6 and 7. What emerges from the simulation results is that HLBO ranks first in optimizing $C_1$, $C_2$, $C_4$, $C_5$, $C_{11}$ to $C_{21}$, $C_{24}$, $C_{26}$, $C_{27}$, $C_{29}$, and $C_{30}$ functions by providing the best performance compared to competitor algorithms. The general analysis of the simulation results of $C_1$ to $C_{30}$ functions shows that HLBO has an acceptable efficiency in handling IEEE CEC 2017 objective functions.

**Table 2:** Evaluation results of unimodal functions.

		GA	PSO	GSA	TLBO	GWO	WOA	TSA	MPA	SMA	HGS	HLBO
$F_1$	Mean	13.391	1.8E−5	2.0E−17	1.3E−59	1.1E−58	1.8E−64	8.2E−33	1.7E−18	0	0	0
	Best	6.905	2E−10	8.2E−18	9.4E−61	7.7E−61	1.3E−65	1.1E−62	3.4E−28	0	0	0
	Std	5.553	5.9E−5	7.1E−18	2.0E−59	4.1E−58	2.8E−64	2.5E−32	6.8E−18	0	0	0
	Med	11.045	9.92E−7	1.8E−17	4.7E−60	1.1E−59	6.3E−65	3.9E−38	1.3E−19	0	0	0
	Rank	9	8	7	3	4	2	5	6	1	1	1
$F_2$	Mean	2.480	0.3412	2.4E−8	5.6E−35	1.3E−34	1.6E−51	5.0E−39	2.8E−9	1.3E−193	8.2E−169	9.3E−222
	Best	1.591	0.0017	1.59E−8	1.3E−35	1.55E−35	1.1E−57	8.3E−43	4.2E−18	0	0	2.3E−223
	Std	0.6428	0.6696	4.0E−9	4.7E−35	2.2E−34	5.9E−51	1.7E−38	1.1E−8	0	0	0
	Med	2.464	0.1301	2.3E−8	4.4E−35	6.4E−35	1.9E−54	8.3E−41	3.2E−11	6.3E−193	0	2.1E−222
	Rank	11	10	9	6	7	4	5	8	2	3	1
$F_3$	Mean	1537.012	589.508	279.358	7.0E−15	7.4E−15	7.6E−9	3.2E−19	0.3770	0	1.0E−143	3.1E−167
	Best	1014.689	1.615	81.912	1.2E−16	4.7E−20	3.4E−9	7.3E−30	0.0320	0	0	6.4E−197
	Std	367.2429	1524.01	112.299	1.3E−14	1.9E−14	2.4E−9	9.9E−19	0.2018	0	4.5E−143	0
	Med	1510.715	54.154	291.532	1.9E−15	1.6E−16	7.2E−9	9.8E−21	0.3787	0	0	1.9E−181
	Rank	11	10	9	5	6	7	4	8	1	3	2
$F_4$	Mean	2.094	3.964	3.3E−9	1.6E−15	1.3E−14	0.0013	2.0E−22	3.7E−8	3.1E−200	2.4E−135	4.8E−206
	Best	1.390	1.605	2.1E−9	6.4E−16	3.4E−16	5.9E−5	1.9E−52	3.4E−17	0	0	9.4E−208
	Std	0.337	2.204	7.5E−10	7.1E−16	2.3E−14	0.0006	6.0E−22	6.5E−8	0	1.1E−134	0
	Med	2.098	3.262	3.4E−9	1.5E−15	7.3E−15	0.0014	3.1E−27	3.0E−8	2.2E−254	0	8.7E−207
	Rank	10	11	7	5	6	9	4	8	2	3	1
$F_5$	Mean	310.452	50.266	36.109	145.675	26.863	27.177	28.770	42.500	5.649	18.018	26.282
	Best	160.501	3.6471	25.838	120.793	25.230	26.451	28.538	41.587	0.0002	7.2E−5	24.770
	Std	120.467	36.525	32.462	19.737	0.882	0.626574	0.365	0.6169	11.104	10.673	0.956
	Med	279.517	28.703	26.075	142.944	26.718	26.935	28.549	42.491	0.2340	23.918	26.538
	Rank	11	9	7	10	4	5	6	8	1	2	3
$F_6$	Mean	14.551	20.2518	0	0.45	0.642	0.071	3.8E−20	0.3909	0.0010	3.2E−7	0
	Best	6.004	5	0	0	1.6E−5	0.0146	6.7E−26	0.2746	0.0005	6.3E−10	0
	Std	5.835	12.7760	0	0.5104	0.3012	0.0782	1.5E−19	0.0803	0.0003	4.1E−7	0
	Med	13.5	19	0	0	0.6215	0.0293	6.7E−21	0.4066	0.0010	2.3E−7	0
	Rank	9	10	1	7	8	5	2	6	4	3	1
$F_7$	Mean	0.0057	0.1134	0.020694	0.0031	0.000819	0.0019	0.0003	0.0022	0.0001	0.0002	0.0001
	Best	0.0021	0.0296	0.01006	0.0014	0.000248	4.2E−5	0.0001	0.0014	3.0E−5	2.2E−6	2.4E−5
	Std	0.0024	0.0459	0.011363	0.0014	0.000503	0.0033	0.0001	0.0005	9.2E−5	0.0003	7.4E−5
	Med	0.0054	0.1079	0.016995	0.0029	0.000629	0.0010	0.0004	0.0022	0.0061	8.7E−5	0.0001
	Rank	9	11	10	8	5	6	4	7	2	3	1
Sum rank		70	69	50	44	40	38	30	51	13	18	10
Mean rank		10	9.8571	7.1429	6.2857	5.4286	5.4286	4.2857	7.2857	1.8571	2.5714	1.4286
Total rank		11	8	8	7	6	5	4	9	2	3	1

**Table 3:** Evaluation results of high-dimensional multimodal functions.

		GA	PSO	GSA	TLBO	GWO	WOA	TSA	MPA	SMA	HGS	HLBO
$F_8$	Mean	− 8184.3	− 6908.6	− 2849.0	− 7803.5	− 5885.1	− 7687.5	− 5669.6	− 3652.1	− 12569.3	− 12569.1	− 8246.4
	Best	− 9717.68	− 8501.4	− 3969.23	− 9103.77	− 7227.05	− 8597.11	− 5706.3	− 4419.9	− 12569.5	− 12569.5	− 8763.3
	Std	795.15	836.7	540.36	986.61	984.50	1105.16	21.86	474.58	0.3973	0.6999	300.44
	Med	− 8117.25	− 7098.95	− 2671.33	− 7735.22	− 5774.63	− 8290.68	− 5669.63	− 3632.65	− 12569.4	− 12569.4	− 8306.6
	Rank	4	7	11	5	8	6	9	10	1	2	3
$F_9$	Mean	62.416	57.065	16.269	10.678	8.5E−15	0	0.0059	152.703	0	0	0
	Best	36.866	27.859	4.975	9.874	0	0	0.0048	128.2306	0	0	0
	Std	15.216	16.517	4.660	0.397	2.0E−14	0	0.0007	15.1857	0	0	0
	Med	61.679	55.225	15.422	10.888	0	0	0.0059	154.621	0	0	0
	Rank	7	6	5	4	2	1	3	8	1	1	1
$F_{10}$	Mean	3.2220	2.154811	3.6E−9	0.2632	1.7E−14	3.9E−15	6.4E−11	8.3E−10	5.1E−15	2.9E−15	1.9E−15
	Best	2.7572	1.155151	2.6E−9	0.1564	1.5E−14	8.9E−16	8.1E−15	1.7E−18	8.9E−16	8.9E−16	8.9E−16
	Std	0.3617	0.549389	5.3E−10	0.0728	3.2E−15	2.6E−15	2.6E−10	2.8E−9	1.5E−14	1.7E−14	1.7E−15
	Med	3.1203	2.170083	3.6E−9	0.2615	1.5E−14	4.4E−15	1.1E−13	1.1E−11	8.9E−16	8.9E−16	8.9E−16
	Rank	11	10	8	9	5	3	6	7	4	2	1
$F_{11}$	Mean	1.2303	0.0463	3.7378	0.5877	0.0038	0.0030	1.6E−6	0	0	0	0
	Best	1.1413	7.3E−9	1.5193	0.3101	0	0	4.2E−15	0	0	0	0
	Std	0.0628	0.0518	1.6703	0.1691	0.0073	0.0135	3.4E−6	0	0	0	0
	Med	1.2272	0.0295	3.4243	0.5820	0	0	8.8E−7	0	0	0	0
	Rank	7	5	8	6	4	3	2	1	1	1	1
$F_{12}$	Mean	0.0470	0.4807	0.0363	0.0206	0.0372	0.0078	0.0502	0.0826	0.0011	6.7E−9	0.0114
	Best	0.0184	0.0001	5.6E−20	0.0020	0.0193	0.0011	0.0354	0.0779	1.9E−5	4.7E−10	0.0036
	Std	0.0285	0.6027	0.0609	0.0286	0.0139	0.0090	0.0099	0.0024	0.0022	6.4E−9	0.0049
	Med	0.0418	0.1556	1.5E−19	0.0152	0.0330	0.0039	0.0509	0.0821	0.0110	0.0004	0.0110
	Rank	8	11	6	5	7	3	9	10	2	1	4
$F_{13}$	Mean	1.2086	0.5084	0.0021	0.3291	0.5764	0.1933	2.6589	0.5653	0.0007	1.3E−7	0.1840
	Best	0.4981	10.0E−7	1.2E−18	0.0383	0.2978	0.0297	2.6318	0.2803	9.8E−6	0.1362	0.1362
	Std	0.3337	1.2517	0.0055	0.1989	0.1704	0.1509	0.0099	0.1878	0.0004	2.0E−7	0.0256
	Med	1.2181	0.0440	2.1E−18	0.2830	0.5783	0.1520	2.6618	0.5799	0.0007	6.4E−8	0.1792
	Rank	10	7	3	6	9	5	11	8	2	1	4
Sum rank		47	46	41	35	35	21	40	44	11	8	14
Mean rank		7.8333	7.6667	6.8333	5.8333	5.8333	3.5	6.6667	7.3333	1.8333	1.3333	2.3333
Total rank		10	9	7	5	5	4	6	8	2	1	3

**Table 4:** Evaluation results of fixed-dimensional multimodal functions.

		GA	PSO	GSA	TLBO	GWO	WOA	TSA	MPA	SMA	HGS	HLBO
$F_{14}$	Mean	0.9987	2.1737	3.5917	2.2644	3.7410	3.1062	1.7988	0.9988	0.9981	1.9746	0.998
	Best	0.9980	0.9980	0.9995	0.9984	0.9980	0.9980	0.9979	0.9981	0.9980	0.9980	0.998
	Std	0.0025	2.9365	2.7792	1.1496	3.9697	3.5336	0.5275	0.0003	0.0002	3.0056	0
	Med	0.9980	0.9980	2.9867	2.2752	2.98217	0.9984	1.9126	0.9989	0.9980	0.9980	0.998
	Rank	3	7	10	8	11	9	5	4	2	6	1
$F_{15}$	Mean	0.0054	0.0017	0.0024	0.0032	0.0064	0.0007	0.0004	0.0039	0.0005	0.0006	0.0003
	Best	0.0008	0.0003	0.0008	0.0022	0.0003	0.0003	0.0003	0.0003	0.0003	0.0003	0.0003
	Std	0.0081	0.0049	0.0012	0.0004	0.0094	0.0003	7.6E−5	0.0051	0.0003	0.0003	4.3E−13
	Med	0.0021	0.0003	0.0023	0.0032	0.0003	0.0005	0.0004	0.0027	0.0004	0.0007	0.0003
	Rank	10	6	7	8	11	5	2	9	3	4	1
$F_{16}$	Mean	− 1.0316	− 1.0316	− 1.0316	− 1.0316	− 1.0316	− 1.0316	− 1.0316	− 1.0316	− 1.0316	− 1.0316	− 1.0316
	Best	− 1.0316	− 1.0316	− 1.0316	− 1.0316	− 1.0316	− 1.0316	− 1.0316	− 1.0316	− 1.0316	− 1.0316	− 1.0316
	Std	4.4E−5	3.2E−5	3.2E−5	3.18E−5	3.2E−5	3.2E−5	3.5E−5	4.1E−5	3.2E−5	3.2E−5	2.2E−16
	Med	− 1.0316	− 1.0316	− 1.0316	− 1.0316	− 1.0316	− 1.0316	− 1.0316	− 1.0316	− 1.0316	− 1.0316	− 1.0316
	Rank	3	2	2	2	2	2	4	4	2	2	1
$F_{17}$	Mean	0.4370	0.7855	0.3979	0.3979	0.3979	0.3979	0.4001	0.4014	0.3979	0.3979	0.3979
	Best	0.3979	0.3979	0.3979	0.3979	0.3979	0.3979	0.3981	0.3989	0.3979	0.3979	0.3979
	Std	0.1407	0.7217	8.6E−5	8.6E−5	8.6E−5	8.6E−5	0.0045	0.0044	8.6E−5	8.6E−5	0
	Med	0.3979	0.3980	0.3979	0.3979	0.3979	0.3979	0.3991	0.3989	0.3979	0.3979	0.3979
	Rank	6	7	2	2	3	3	4	5	2	2	1
$F_{18}$	Mean	4.3605	3.0002	3.0002	3.0002	3.0002	3.0002	3.0018	3.0002	3.0002	3.	3.
	Best	3.	3.	3.	3.	3.	3.	3.	3.	3.	3.	3
	Std	6.0399	0.0006	0.0006	0.0006	0.0006	0.0006	0.0009	0.0006	0.0006	0.0006	1.1E−15
	Med	3.0011	3.	3.	3.	3.0000	3.0000	3.0018	3.	3.	3.	3.
	Rank	6	2	2	2	4	3	5	2	2	2	1
$F_{19}$	Mean	− 3.8543	− 3.8627	− 3.8627	− 3.8613	− 3.8621	− 3.8606	− 3.8066	− 3.8627	− 3.8627	− 3.8627	− 3.8628
	Best	− 3.8628	− 3.8628	− 3.8628	− 3.8625	− 3.8628	− 3.8628	− 3.8366	− 3.8627	− 3.8627	− 3.8627	− 3.8628
	Std	0.0148	0.0001	0.0001	0.0014	0.0017	0.0029	0.0153	0.0002	0.0002	0.0002	1.4E−7
	Med	− 3.8624	− 3.8628	− 3.8628	− 3.862	− 3.8628	− 3.8617	− 3.8066	− 3.8627	− 3.8627	− 3.8627	− 3.8628
	Rank	7	2	2	5	4	6	8	3	2	2	1
$F_{20}$	Mean	− 2.8239	− 3.2619	− 3.3220	− 3.2011	− 3.2523	− 3.2229	− 3.3195	− 3.3211	− 3.2386	− 3.2804	− 3.3220
	Best	− 3.3134	− 3.322	− 3.322	− 3.2617	− 3.3220	− 3.3220	− 3.3212	− 3.3213	− 3.3220	− 3.3220	− 3.3220
	Std	0.3860	0.0706	0.0001	0.0318	0.0766	0.0904	0.0031	0.0001	0.0560	0.0582	2.8E−5
	Med	− 2.9683	− 3.3217	− 3.322	− 3.2076	− 3.2623	− 3.1952	− 3.3206	− 3.3211	− 3.2031	− 3.3220	− 3.3220
	Rank	11	6	2	10	7	9	4	3	8	5	1
$F_{21}$	Mean	− 4.6040	− 5.5392	− 5.4486	− 9.1901	− 9.4451	− 8.8763	− 5.5020	− 9.9043	− 10.153	− 9.8982	− 10.1532
	Best	− 8.5213	− 10.1532	− 10.1532	− 9.6639	− 10.1532	− 10.1531	− 9.5021	− 10.1532	− 10.1532	− 10.1532	− 10.1532
	Std	1.9247	3.0763	3.0940	0.1207	1.7395	2.2635	1.2566	0.5592	0.0004	1.1400	4.2E−10
	Med	− 4.3747	− 5.1008	− 3.7693	− 9.1532	− 10.1525	− 10.1512	− 5.5021	− 10.1532	− 10.1531	− 10.1532	− 10.1532
	Rank	11	8	10	6	5	7	9	3	2	4	1
$F_{22}$	Mean	− 5.1174	− 7.6322	− 9.7664	− 10.0486	− 10.4024	− 9.3372	− 5.9134	− 10.2858	− 10.4027	− 10.4028	− 10.4029
	Best	− 9.1106	− 10.4029	− 10.4029	− 10.4029	− 10.4028	− 10.4028	− 9.0625	− 10.4029	− 10.4029	− 10.4029	− 10.4029
	Std	1.9696	3.5417	1.7084	0.3983	0.0004	2.1800	1.7549	0.2454	0.0003	0.0003	1.9E−5
	Med	− 5.0294	− 10.4024	− 10.4029	− 10.1836	− 10.4025	− 10.4012	− 5.0628	− 10.4028	− 10.4028	− 10.4029	− 10.4029
	Rank	11	9	7	6	4	8	10	5	3	2	1
$F_{23}$	Mean	− 6.5621	− 6.1647	− 10.0188	− 9.2642	− 10.1302	− 9.4522	− 9.8098	− 10.1408	− 10.5362	− 10.2659	− 10.5364
	Best	− 10.2216	− 10.5364	− 10.5364	− 10.534	− 10.5363	− 10.5363	− 10.3683	− 10.5364	− 10.5364	− 10.5364	− 10.5364
	Std	2.6172	3.7349	1.5938	1.6765	1.8144	2.2219	1.6064	1.1401	0.0003	1.2094	5.4E−6
	Med	− 6.5629	− 4.5055	− 10.5364	− 9.6717	− 10.536	− 10.535	− 10.3613	− 10.5364	− 10.5363	− 10.5364	− 10.5364
	Rank	10	11	6	9	5	8	7	4	2	3	1
Sum rank		78	60	50	58	56	60	58	42	28	32	10
Mean rank		7.8	6	5	5.8	5.6	6	5.8	4.2	2.8	3.2	1
Total rank		9	8	5	7	6	8	7	4	2	3	1

## Results and discussion

Optimization algorithms by utilizing exploration for global search and exploitation for local search, have the ability to handle optimization problems. To analyze the exploitation ability of HLBO in local search, the unimodal objective functions are favorable with only one main peak. In this type of optimization issues, the main challenge is the convergence towards the global optima. The optimization results of unimodal functions using HLBO indicate the exploitation ability of the proposed method in converging to the global optimal solution. In particular, HLBO has demonstrated its high local search ability by converging to the global optimal in handling the functions $F_1$ and $F_6$. High-dimensional multimodal functions due to the existence of multiple local optimal solutions are a suitable option for measuring the exploratory ability of optimization algorithms for global search and finding the main optimal area. The main challenge in solving these problems is to accurately scan the search space and prevent the algorithm from getting stuck in some of the optimal local areas. The results of implementing HLBO on high-dimensional multimodal functions show that the proposed approach has an acceptable exploration ability in scanning the search space and finding the optimal area. The exploratory power of HLBO in identifying the optimal region, especially in the $F_9$ and $F_{11}$ functions, is evident that it has been able to provide the global optimal. In addition to having the right quality of exploration and exploitation, having the right balance between these two indicators is the key to the success of optimization algorithms. Fixed dimensional multimodal functions have been selected to evaluate the ability of HLBO to strike a balance between exploration and exploitation. In this type of problem, it is important to simultaneously find the main optimal area based on global search and converge as much as possible to the global optimal based on local search. The optimization results of this type of function using the proposed approach show the high capability of HLBO in balancing exploration and exploitation to discover the optimal area and converge towards the global optimal.

**Table 5:** p-values results from Wilcoxon sum rank test.

Compared algorithms	Test function type		
	Unimodal	High-multimodal	Fixed-multimodal
HLBO versus SMA	0.000330083	1.63352E−12	0.432505732
HLBO versus HGS	3.09811E−6	1.63352E−12	9.93431E−30
HLBO versus MPA	2.73907E−22	0.000331172	4.9571E−34
HLBO versus TSA	3.24444E−7	6.60407E−10	3.6466E−34
HLBO versus WOA	2.03592E−7	0.016139556	1.43615E−34
HLBO versus GWO	0.000121056	0.844919773	1.43615E−34
HLBO versus TLBO	4.24099E−22	0.001091824	1.49171E−25
HLBO versus GSA	3.3289E−8	6.10009E−8	0.001848864
WOA versus PSO	9.54457E−19	8.18756E−10	5.68697E−9
WOA versus GA	1.03289E−24	2.05428E−6	1.43615E−34

**Table 6:** Evaluation results of IEEE CEC 2017 objective functions $C_1$ to $C_{18}$.

		GA	PSO	GSA	TLBO	GWO	WOA	TSA	MPA	SMA	HGS	HLBO
$C_{1}$	Avg	9800	3960	296	2.0E+7	3.3E+5	8.5E+6	296	3400	156	2470	100
	Std	6210	4660	287	4.2E+6	1.1E+5	2.4E+7	287	3840	3.8E+4	277	501
	Rank	7	6	3	10	8	9	3	5	2	4	1
$C_{2}$	Avg	5610	7060	7910	1.2E+4	314	461	216	219	201	201	200
	Std	4360	2290	2260	6660	7510	7380	797	701	77.9	99	10.9
	Rank	7	8	9	10	5	6	3	4	2	2	1
$C_{3}$	Avg	8720	300	1.1E+4	2.8E+4	1540	2.3E+4	1.1E+4	300	301	1510	300
	Std	6170	2.0E−10	1690	9240	1980	3900	1670	0	50.1	26.5	1.0E−10
	Rank	5	1	6	8	4	7	6	1	2	3	1
$C_{4}$	Avg	411	406	407	548	410	2390	407	406	403	404	400
	Std	19.3	3.43	3.05	15.9	7.89	431	3.05	10.6	99	8.54	0.0596
	Rank	7	4	5	8	6	9	5	4	2	3	1
$C_{5}$	Avg	516	513	557	742	514	900	557	522	530	513	510
	Std	7.24	6.83	8.78	36.9	6.37	83.1	8.79	11	60.9	25.4	4.13
	Rank	4	2	7	8	3	9	7	5	6	2	1
$C_{6}$	Avg	600	600	622	665	601	691	622	610	682	600	600
	Std	0.698	1.02	9.43	43.9	0.092	11.4	9.43	8.63	37	1.46	6.4E−4
	Rank	1	1	4	5	2	7	4	3	6	1	1
$C_{7}$	Avg	728	719	715	1280	730	1860	715	741	713	713	723
	Std	7.62	5.33	1.62	44.1	8.99	79.8	1.63	17.3	1.7	4.49	4.09
	Rank	5	3	2	8	6	9	2	7	1	1	4
$C_{8}$	Avg	821	811	821	952	814	1070	821	823	829	809	809
	Std	9.36	5.72	4.9	19.9	8.63	46.5	4.9	10.4	55.4	8.37	3.26
	Rank	5	3	5	8	4	9	5	6	7	1	2
$C_{9}$	Avg	910	900	900	6800	911	2.9E+4	900	944	4670	910	900
	Std	15.9	6.2E−14	6.2E−15	1360	20.4	9130	0	110	2150	20.9	0.017
	Rank	2	1	1	6	3	7	1	4	5	2	1
$C_{10}$	Avg	1720	1470	2690	5290	1530	7470	2690	1860	2590	1410	1440
	Std	263	225	311	674	300	1420	311	308	433	36.6	147
	Rank	5	3	8	9	4	10	8	6	7	1	2
$C_{11}$	Avg	1130	1110	1130	1270	1140	1920	1130	1180	1110	1110	1100
	Std	24.9	6.56	11	41.6	56.5	1980	11	62.5	26.5	11.7	1.33
	Rank	3	2	3	6	4	7	3	5	2	2	1
$C_{12}$	Avg	3.7E+4	1.4E+4	7.0E+5	2.2E+7	6.3E+5	1.8E+8	7.1E+5	2.0E+6	1630	1.5E+4	1250
	Std	3.6E+4	1.2E+4	4.4E+4	2.2E+7	1.2E+6	1.8E+9	4.4E+5	2.0E+6	207	2800	56.7
	Rank	5	3	7	10	6	11	8	9	2	4	1
$C_{13}$	Avg	1.1E+4	8600	1.1E+4	4.2E+5	9840	1.9E+8	1.1E+4	1.61E+4	1320	6820	1310
	Std	9330	5350	2200	1.3E+5	5880	1.4E+8	2200	1.1E+4	81.8	4450	2.7
	Rank	6	4	7	9	5	10	7	8	2	3	1
$C_{14}$	Avg	7050	1480	7150	4.1E+5	3400	2.0E+6	7150	1510	1450	1450	1400
	Std	8530	44.4	1560	2.4E+5	2040	7.3E+6	1560	53.4	58.5	23.4	4.24
	Rank	6	3	7	8	5	9	7	4	2	2	1
$C_{15}$	Avg	9300	1710	1.8E+4	4.8E+4	3810	1.4E+7	1.8E+4	2240	1510	1580	1500
	Std	9380	296	5750	1.6E+4	4030	2.1E+7	5750	597	17.1	134	0.543
	Rank	7	4	8	9	6	10	8	5	2	3	1
$C_{16}$	Avg	1790	1860	2150	3500	1730	3000	2150	1730	1820	1730	1600
	Std	135	134	111	239	130	1250	111	133	240	125	1.03
	Rank	3	5	6	8	2	7	6	2	4	2	1
$C_{17}$	Avg	1750	1760	1860	2630	1760	4340	1860	1770	1830	1730	1710
	Std	41.6	49.6	113	199	32.7	331	113	35.7	184	36.1	9.86
	Rank	3	4	7	8	4	9	7	5	6	2	1
$C_{18}$	Avg	1.6E+4	1.5E+4	8720	7.5E+5	2.6E+4	3.7E+7	8720	2.3E+4	1830	7440	1800
	Std	1.3E+4	1.2E+4	5290	3.9E+5	1.6E+4	5.2E+7	5290	1.5E+4	14.1	4720	0.543
	Rank	6	5	4	9	8	10	4	7	2	3	1

**Table 7:** Evaluation results of IEEE CEC 2017 objective functions $C_{19}$ to $C_{30}$.

		GA	PSO	GSA	TLBO	GWO	WOA	TSA	MPA	SMA	HGS	HLBO
$C_{19}$	Avg	9690	2600	1.4E+4	6.1E+5	9870	2.3E+6	4.5E+4	2920	1920	1950	1900
	Std	7070	2290	2.0E+4	5.7E+5	6660	1.7E+7	2.0E+4	1950	30	57.8	0.47
	Rank	6	4	8	10	7	11	9	5	2	3	1
$C_{20}$	Avg	2060	2090	2270	2870	2080	3790	2270	2090	2490	2020	2020
	Std	62.7	65.1	85.4	213	54.3	462	85.4	51.5	254	26.4	10.1
	Rank	3	5	6	8	4	9	6	5	7	2	1
$C_{21}$	Avg	2300	2280	2360	2580	2320	2580	2360	2250	2320	2230	2200
	Std	45.8	56.4	29.5	64.5	7.32	192	29.5	63.1	70.9	45.5	21.3
	Rank	5	4	7	8	6	8	7	3	6	2	1
$C_{22}$	Avg	2300	2310	2300	7180	2310	1.4E+4	2300	2310	3530	2280	2280
	Std	2.49	69.1	0.0752	1340	17.6	1080	0.0732	12.3	886	13.9	39.8
	Rank	3	4	3	6	4	7	3	3	5	1	2
$C_{23}$	Avg	2630	2620	2740	3120	2620	3810	2740	2620	2730	2610	2610
	Std	14	9.65	40.9	86.8	8.85	229	4.9	9.08	254	4.31	4.11
	Rank	4	3	6	7	3	8	6	3	5	1	2
$C_{24}$	Avg	2760	2690	2740	3330	2740	3480	2740	2730	2700	2620	2520
	Std	15.6	131	5.77	169	9.12	229	5.8	67.3	76.7	83.2	40.1
	Rank	7	3	6	8	6	9	6	5	4	2	1
$C_{25}$	Avg	2950	2920	2940	2910	2940	3910	2940	2920	2930	2920	2900
	Std	20.2	26.1	16.1	18.4	24.7	266	16	25	21.8	13.2	0.512
	Rank	6	3	5	2	5	7	5	3	4	3	1
$C_{26}$	Avg	3110	2950	3.4E+4	7870	3230	7100	3440	2900	3460	3110	2850
	Std	350	261	657	951	2970	657	38.2	626	302	23.4	101
	Rank	4	3	10	9	5	8	6	2	7	4	1
$C_{27}$	Avg	3120	3120	3260	3410	3100	4810	3260	3090	3140	3110	3090
	Std	20.1	26.1	43.6	85.8	22.8	642	43.6	2.91	22.4	21.8	0.481
	Rank	5	5	7	8	3	9	7	2	6	4	1
$C_{28}$	Avg	3320	3320	3460	3400	3390	5090	3460	3210	3400	2300	3100
	Std	132	127	35.3	124	107	329	35.3	118	137	130	6.6E−5
	Rank	4	4	7	6	5	8	7	3	6	1	2
$C_{29}$	Avg	3250	3200	3450	4560	3190	8890	3450	3210	3210	3210	3150
	Std	85.7	84.7	179	516	44.8	1480	179	54	115	59.1	134
	Rank	5	3	6	7	2	8	6	4	4	4	1
$C_{30}$	Avg	5.4E+5	3.5E+5	1.3E+6	4.0E+6	3.0E+5	1.9E+7	9.4E+5	4.2E+5	3.1E+5	3.0E+5	3410
	Std	6.7E+5	5.3E+5	3.8E+5	1.7E+6	5.5E+5	1.4E+8	3.8E+5	5.9E+5	4.7E+5	2.2E+4	28
	Rank	7	5	9	10	3	11	8	6	4	2	1
Sum rank		146	108	179	236	138	258	170	134	122	70	38
Mean rank		0.1856	0.1422	0.2289	0.3133	0.1844	0.3389	0.2411	0.1767	0.1578	0.0978	0.0489
Total rank		7	3	9	10	6	11	8	5	4	2	1

## Conclusion and future works

In this paper, a new optimization algorithm called Hybrid Leader Optimization (HLBO) is introduced. The use of a hybrid leader generated by three different members was HLBO’s idea in updating the algorithm population in the search space. The HLBO implementation process was mathematically modeled in two phases of exploration and exploitation. Twenty-three objective functions were employed to evaluate the performance of HLBO in achieving optimal solutions for optimization problems. The results of the unimodal functions indicated the high exploitation ability of HLBO to search locally and converge towards global optima. The results of optimizing multimodal functions showed the high exploration ability of HLBO to search globally and discover the optimal area without getting caught up in local optimal. For further analysis of HLBO, its efficiency in handling complex IEEE CEC 2017 objective functions was studied. The results showed that HLBO is capable of solving such optimization problems.

The results of HLBO compared with the performance of ten well-known algorithms showed that HLBO has a superior performance by providing appropriate solutions in most cases due to the appropriate balance between exploration and exploitation. The proposed HLBO opens up several research subjects for further work in the future. Specific research potentials are the development of binary and multimodal versions of HLBO. The HLBO employment on optimization topics in various sciences as well as real-world applications are other suggestions for future studies. Similar to any stochastic-based optimization algorithm, there are concerns and limitations for the use of the proposed HLBO approach. Of course, we do not claim that HLBO is generally the best optimizer because according to the NFL theorem, there is no presupposition for the effective performance of an algorithm in dealing with optimization issues. It is also possible that there may be other algorithms or that new algorithms may be developed by researchers in the future that work better in some concrete applications.

## Data Availability

All data generated or analysed during this study are included in this published article [and its supplementary information files].
